# Differential kynurenine pathway metabolism in highly metastatic aggressive breast cancer subtypes: beyond IDO1-induced immunosuppression

**DOI:** 10.1186/s13058-020-01351-1

**Published:** 2020-10-27

**Authors:** Benjamin Heng, Ayse A. Bilgin, David B. Lovejoy, Vanessa X. Tan, Heloisa H. Milioli, Laurence Gluch, Sonia Bustamante, Tharani Sabaretnam, Pablo Moscato, Chai K. Lim, Gilles J. Guillemin

**Affiliations:** 1 Department of Biomedical Sciences, Faculty of Medicine and Health Sciences, Macquarie University, Sydney, Australia; 2 Faculty of Sciences and Engineering, Macquarie University, Sydney, Australia; 3 School of Environmental and Life Sciences, The University of Newcastle, Callaghan, Australia; 4 The Strathfield Breast Centre, Strathfield, Australia; 5 Bioanalytical Mass Spectrometry Facility, University of New South Wales, Sydney, Australia; 6 School of Electrical Engineering and Life Sciences, The University of Newcastle, Callaghan, Australia

**Keywords:** Kynurenine pathway, Breast cancer, Biomarker, Immune evasion, Tryptophan

## Abstract

**Background:**

Immunotherapy has recently been proposed as a promising treatment to stop breast cancer (BrCa) progression and metastasis. However, there has been limited success in the treatment of BrCa with immune checkpoint inhibitors. This implies that BrCa tumors have other mechanisms to escape immune surveillance. While the kynurenine pathway (KP) is known to be a key player mediating tumor immune evasion and while there are several studies on the roles of the KP in cancer, little is known about KP involvement in BrCa.

**Methods:**

To understand how KP is regulated in BrCa, we examined the KP profile in BrCa cell lines and clinical samples (*n* = 1997) that represent major subtypes of BrCa (luminal, HER2-enriched, and triple-negative (TN)). We carried out qPCR, western blot/immunohistochemistry, and ultra-high pressure liquid chromatography on these samples to quantify the KP enzyme gene, protein, and activity, respectively.

**Results:**

We revealed that the KP is highly dysregulated in the HER2-enriched and TN BrCa subtype. Gene, protein expression, and KP metabolomic profiling have shown that the downstream KP enzymes KMO and KYNU are highly upregulated in the HER2-enriched and TN BrCa subtypes, leading to increased production of the potent immunosuppressive metabolites anthranilic acid (AA) and 3-hydroxylanthranilic acid (3HAA).

**Conclusions:**

Our findings suggest that KMO and KYNU inhibitors may represent new promising therapeutic targets for BrCa. We also showed that KP metabolite profiling can be used as an accurate biomarker for BrCa subtyping, as we successfully discriminated TN BrCa from other BrCa subtypes.

## Background

Breast cancer (BrCa) metastasis is the leading cause of cancer-related death in women [1, 2], indicating the need for new therapeutic targets. Immune checkpoint inhibitors have recently emerged as a new approach which enables immune cells to recognize and destroy cancer cells. In some metastatic tumors such as melanoma, immune checkpoint inhibitors have demonstrated significant therapeutic potential [3]. Despite BrCa often presenting with infiltrated immune cells [4–6], the efficacy of current immune checkpoint inhibitors in BrCa is far from optimal, suggesting the possibility of other dominant mechanisms that prevent or limit the immune response towards BrCa.

**Fig. 1:**
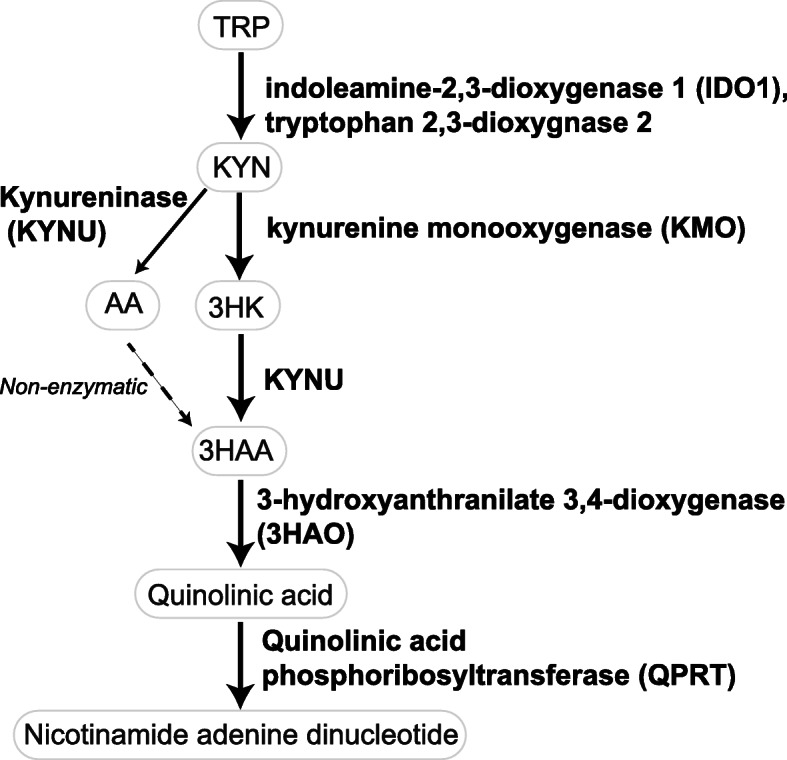
A simplified diagram of the KP. Majority of TRP in the body is catabolized through KP to synthesize the vital energy cofactor, nicotinamide adenine dinucleotide. This pathway is frequently elevated in the inflammatory environment such as cancer and has been shown to promote immune evasion. This immune modulation results from the overactivation of IDO1/trytophan 2,3-dioxygenase 2 that leads to the depletion of local TRP, an essential amino acid for T cell proliferation, and production of bioactive metabolites (KYN, 3HK, AA, and 3HAA) that promotes selective apoptosis of tumor-targeting T cells

Considering the strong evidence linking the kynurenine pathway (KP) to immunosuppression and tumor growth, the KP has been identified as a key immunotherapeutic cancer target [7, 8] (Fig. 1). Through induction of its first rate-limiting enzyme, indoleamine-2,3-dioxygenase 1 (IDO1), the KP has been associated with poorer prognosis in cancer patients [9, 10]. Using a murine model, Uyttenhove et al. showed that a tumor with elevated IDO1 activity was not detected nor destroyed by tumor-specific host immune cells [11]—a phenomenon shown to result from tryptophan (TRP) depletion by the IDO1-expressing tumor. Later studies also revealed that other bioactive KP metabolites may have immune-modulating properties. Fallarino et al. and Zaher et al. demonstrated that downstream KP metabolites such as 3-hydroxykynurenine (3HK), 3-hydroxyanthranilic acid (3HAA), and quinolinic acid can significantly inhibit T cell proliferation and induce T cell apoptosis [12, 13], thereby highlighting the important roles of other KP metabolites in affecting immune cells.

Despite the growing interest in the KP and cancers, there are limited studies examining the role of KP in human BrCa. Previous studies have shown elevated expression of IDO1 mRNA and protein in BrCa cells, as well as evidence of elevated IDO1 activity in BrCa patient sera compared to healthy controls [14–18]. Evidence also suggests that the KP may be involved in BrCa metastasis, as BrCa patients with lymph node metastasis showed increased regulatory T cells and a higher density of IDO1-expressing macrophages in their lymph nodes [19]. A separate study supported these findings, reporting that BrCa patients with bone metastases had elevated IDO1 activity in the sera, compared to patients without metastases [20]. Although there is strong accumulating evidence that IDO1 overactivation is involved in human BrCa, a significant limitation of these studies is that the KP profiling data has been limited to characterizing the degradation of TRP to its intermediate, kynurenine (KYN) by the enzymes IDO1 or tryptophan 2,3-dioxygenase 2.

The potential roles of other KP enzymes and downstream KP metabolites remain totally unexplored in BrCa. Considering that some of these are known to be immunomodulatory, this represents a considerable knowledge gap. Also, whether KP enzymes and metabolites differ between BrCa subtypes (i.e., luminal, HER2-enriched, and triple-negative (TN)) and how these differences could be related to the differences in metastatic potential and disease aggressiveness is still unknown. Further, the KP enzyme kynurenine monooxygenase (KMO), active downstream of IDO1, has been shown to play a critical role in human hepatocellular carcinoma [21] and to be independent of IDO1 activity. Hence, it is important to examine how KP is regulated in each subtype to understand which KP enzyme is involved in the development and/or progression of BrCa. The objectives of this study were to fully characterize the activity of the KP in each BrCa subtype and to investigate whether variations in serum KP parameters are associated with particular BrCa clinical subtypes.

## Methods

### Human BrCa cell lines

Human breast cancer cell lines representing the three major subtypes luminal BrCa cell lines (MCF7 and T47D), TN BrCa cell lines (MDA-MB-231, MDA-MB-468, MDA-MB-157, and HBL100), and HER2-enriched BrCa cell line (SK-BR3) were cultured in RPMI (Life Technologies) containing 10% fetal calf serum and 1% antibiotic and antimycotic and were maintained at 37 °C with 5% CO_2_ in a humidified atmosphere. Cell lines were authenticated by the Garvan Institute of Medical Research using short tandem repeat DNA profiling and were found to be > 93% concordant.

### The Molecular Taxonomy of Breast Cancer International Consortium (METABRIC) cohort

The data used in this study consists of transcriptomic (cDNA microarray) information processed using the Illumina HT-12 v3 platform (Illumina_Human_WG-v3). Gene expression values of primary breast tumors were extracted from luminal (1140 samples), HER2-enriched (220 samples), and TN (199 samples) subtypes and from healthy control (HC) tissues (144 samples) [22].

### Patient cohort

Two cohorts of BrCa clinical samples used in this study were sourced from the Victoria Cancer Biobank consortium, the Australian Breast Cancer Tissue Bank, or the Strathfield Breast Centre. Cohort 1 comprised 506 serum samples from 408 BrCa patients with luminal, HER2-enriched, and TN subtype and 98 HC. Cohort 2 consisted of 30 formalin-fixed tumor tissues from TN, HER2-enriched, or luminal subtype BrCa patients. As shown in Table 1, the groups were well distributed in both cohorts. The mean age differences between the BrCa groups were well controlled except for HC which was significantly younger (*p* < 0.0001).

**Table 1:** Demographic and clinical characteristics of cohorts 1 and 2

Subtype	Cohort 1				Cohort 2		
	HC (%)	Luminal (%)	TN (%)	HER2-enriched (%)	Luminal (%)	TN (%)	HER2-enriched (%)
Total	98	138	143	127	10	10	10
Age							
Mean	44.99	61.3	57.3	59.6	66	64.1	62.1
< 40	33 (33.6)	5 (3.6)	17 (11.9)	6 (4.7)	1 (10)	0	0
40–49	26 (26.5)	18 (13.0)	24 (16.8)	25 (19.6)	0	1 (10)	2 (20)
50–59	24 (24.5)	43 (31.2)	37 (25.9)	34 (26.8)	3 (30)	2 (20)	3 (30)
60–69	14 (14.3)	37 (26.8)	31 (21.7)	31 (24.4)	1 (10)	3 (30)	3 (30)
> 70	1 (1.0)	35 (25.3)	34 (23.7)	31(24.4)	5 (50)	4 (40)	2 (20)
Histological grade							
1		34 (24.6)	0	0	0	0	0
2		62 (44.9)	52 (36.4)	34 (26.8)	0	0	0
3		42 (30.4)	91 (63.6)	93 (73.2)	10 (100)	10 (100)	10 (100)
Tumor size							
< 2 cm		58 (42.0)	68 (47.6)	43 (33.8)	2 (20)	2 (20)	1 (10)
2.0–5.0 cm		57 (41.3)	72 (50.3)	65 (51.2)	5 (50)	8 (80)	8 (80)
> 5 cm		23 (16.7)	3 (2.1)	18 (14.2)	3 (30)	0	1 (10)
Not available		0	0	1 (0.8)	0	0	0
PR status							
Positive		138 (100)	0 (0)	0 (0)	8 (80)	0 (0)	12 (9.4)
Negative		0 (0)	143 (100)	127 (100)	2 (20)	10 (100)	115 (90.6)
HER2 IHC							
Positive		119 (86.2)	0 (0)	12 (9.4)	0 (0)	0 (0)	10 (100)
Negative		19 (13.8)	143 (100)	115 (90.6)	10 (100)	10 (100)	0 (0)

All samples used in this study were from female patients who were diagnosed with primary BrCa as their first cancer event. Blood was collected before surgery, and tumor tissues were collected before chemotherapy treatment. Estrogen, progesterone receptor, and HER2 status were determined by qualified pathologists using immunohistochemistry. HC sera were sourced from the Australian Breast Cancer Tissue Bank.

### mRNA extraction and qPCR

To determine the gene expression along the KP, total mRNA was extracted with the RNeasy Mini Kit (Qiagen) according to the manufacturer’s instructions. Following extractions, the quantity and quality of the total mRNA were measured using the Nanodrop 2000 (Thermo Fisher Scientific). For cDNA synthesis, 2 μg of total mRNA was reverse transcribed with Superscript VILO cDNA Synthesis Kit (Thermo Fisher Scientific) according to the manufacturer’s instructions. qPCR reactions were performed in a final volume of 10 μl with each reaction mix containing 5 μl Fast SYBR® green master mix, 5 μM forward and reverse primers, and 125 ng of cDNA template in the Viia7 (Thermo Fisher Scientific). The reaction was incubated at 95 °C for 20 s, then amplified for 40 cycles of 95 °C for 1 s and 60 °C for 20 s. A melting curve was generated at the end of each reaction to confirm that only one product was formed. The mRNA expression levels of KP genes were normalized to tubulin-binding protein (TBP) and made relative to the untreated control condition using the 2^−ΔΔC^_T_ method*.* The sequences and efficiency of qPCR primers are generated in accordance with the MIQE PCR Guidelines [23] and are shown in Supplementary Table 1.

### Protein lysate preparation and western blot assay

Cells were plated to achieve 70% confluency and treated for 48 h with IFN-γ (specific activity, 1 × 10^7^ IU/mg; Miltenyi Biotech) or RPMI media as control. The cells were then lysed in a buffer containing 20 mM Tris-HCL (pH 8.0), 137 mM NaCl, 1% NP40, 10% glycerol, and 1× Protease Inhibitor Cocktail (Promega). Protein concentrations were measured by the Pierce™ BCA protein assay kit (Thermo Fisher Scientific). NuPAGE® sample reducing agent (Thermo Fisher Scientific) and Laemmli buffer (BioRad) were added to the samples and heated to 70 °C for 10 min. Denatured samples were transferred onto ice before separation by electrophoresis on a kD™ Mini-PROTEAN® TGX protein gel (BioRad). The proteins were then transferred to nitrocellulose membranes and blocked with 5% skim milk for an hour. Blots were probed overnight at 4 °C with primary antibodies: IDO1 (1:1000; clone: UMAB126, Origene), KMO (1:1000; LSBio), kynureninase (KYNU) (1:500; clone: OTI1H1, Origene), and actin (1:1000; Abcam). Secondary anti-mouse (1:10,000; Dako) and anti-rabbit (1:12,000; Dako) antibodies were incubated for its corresponding primary antibody for an hour before developing with Clarity™ Western ECL substrate (Bio-Rad).

### Quantification of KP metabolites

Prior to analysis, 150 μl of biological fluids was deproteinized with 10% (w/v) trichloroacetic acid in equal proportions. Samples were incubated for 5 min, vortexed, then centrifuged (4 °C) for 10 min at 12,000 rpm. The supernatant was then extracted and filtered with 0.22-μm syringe filters (Millex, Merck) ready for injection into the analyzers.

Concurrent quantification of TRP, KYN, 3HK, 3HAA, and AA was carried out as previously described [24]. Briefly, 20 μl of the filtered extract was injected into the analyzer. Separation of metabolites was performed under a stable temperature of 38 °C for 12 min, using 0.1 mM sodium acetate (pH 4.65) as the mobile phase, with an isocratic flow rate of 0.75 ml/min in an Eclipse Plus C18 reverse-phase column (2.1 mm × 150 mm, 1.8 μm particle size, Agilent). 3HK and KYN were detected using UV wavelength at 365 nM. TRP, 3HAA, and AA were detected using fluorescence intensity set at Ex/Em wavelength of 280/438 for TRP and 320/438 for 3HAA and AA. Mixed standards of all metabolites were used for a 6-point calibration curve in order to interpolate the quantity of the sample readout. Agilent OpenLAB CDS Chemstation (Edition C.01.04) was used to analyze the chromatogram. The inter- and intra-assay coefficient of variation is within the acceptable range of 3–7%. Concentrations of KP metabolites in cell culture media were calculated by subtracting the values of pre- and post-treatment concentrations.

### Immunohistochemistry and scoring of staining

Formalin-fixed paraffin-embedded tissue sections (8 μM) were purchased from the Victoria Cancer Biobank. The sections were deparaffinized and rehydrated through graded alcohols to water. Antigen retrieval was performed by boiling the de-paraffinized sections in specific buffers according to each antibody. After placing the slides onto a chamber stacker, they were rinsed thrice with a wash buffer (Dako). Endogenous peroxidase activity was blocked with a 10-min incubation of Dual Endogenous Enzyme-Blocking Reagent (Dako). Thereafter, the slides were rinsed with wash buffer (Dako) and blocked with 5% BSA (Sigma Aldrich) in PBS-T (PBS with 0.2% Tween20) for 1 h at room temperature. The primary KP enzyme antibodies (IDO1 1:100 and KYNU 1:100 antibody as mentioned above in western blot; KMO, 1:100 (Sigma Aldrich) and isotype control antibody (IDO1 isotype control IgG1/clone DAK-GO1 (Dako), KMO isotype control rabbit IgG (Abcam), KYNU isotype control IgG2b/clone DAK-GO9 (Dako) were applied overnight at 4 °C. After primary antibody incubation, the sections were washed and incubated for 1 h with a peroxidase-labeled secondary antibody specific for each primary antibody.

The slides were scored numerically by three blinded researchers, and a composite staining score was calculated based on two categories: (1) the percentage of tumor stained positive (0 = 0%, 1 = 1–33%, 2 = 34–66%, 3 = > 66%) and (2) intensity of protein staining (0, 1, 2, 3). Differences in scores were adjudicated between the researchers to arrive at a final score.

### Statistical analysis and modeling

Descriptive statistics were used to identify outliers, missing data, and normality of KP variables and demographics. Where needed, data normalization was performed prior to analysis. Exploratory data analysis involving multiple groups or case-control comparison was performed using one-way ANOVA and *t* test, respectively. Differences in the expression/level of variables of interests were considered significant if *p* < 0.05.

To develop an algorithm that can potentially discriminate the BrCa subtypes based on predictors (i.e., variables of interests identified during exploratory analysis), a supervised machine learning approach using various classification models was applied. These models included the Classification and Regression Tree, neural networks [25], support vector machines, discriminant analysis, and C5.0 decision tree [26, 27] that were previously described for a similar study design [24]. First, we randomly split the dataset into training (77%) and test (23%) sets. Then, an iterative model-building framework approach was used to find the best model for our aim. A classification model was considered successful when it had the highest predictive accuracy for specific subtype observations. In addition to accuracy, we calculated the class-specific lifts, sensitivity, and specificity of the models. To minimize the overfitting of the model, a 10-fold cross-validation and pruning set at 75% were implemented during the analysis.

All statistical analyses were performed using the *R* software (R Core team 2015), with the *R* package [28] and illustrated with Prism 8 (GraphPad) and Excel. All classification modeling was developed using IBM SPSS Modeler (version 18.0, 2016).

## Results

### IDO1 expression and activity is predominately upregulated in TN and HER2-enriched BrCa subtype

**Fig. 2:**
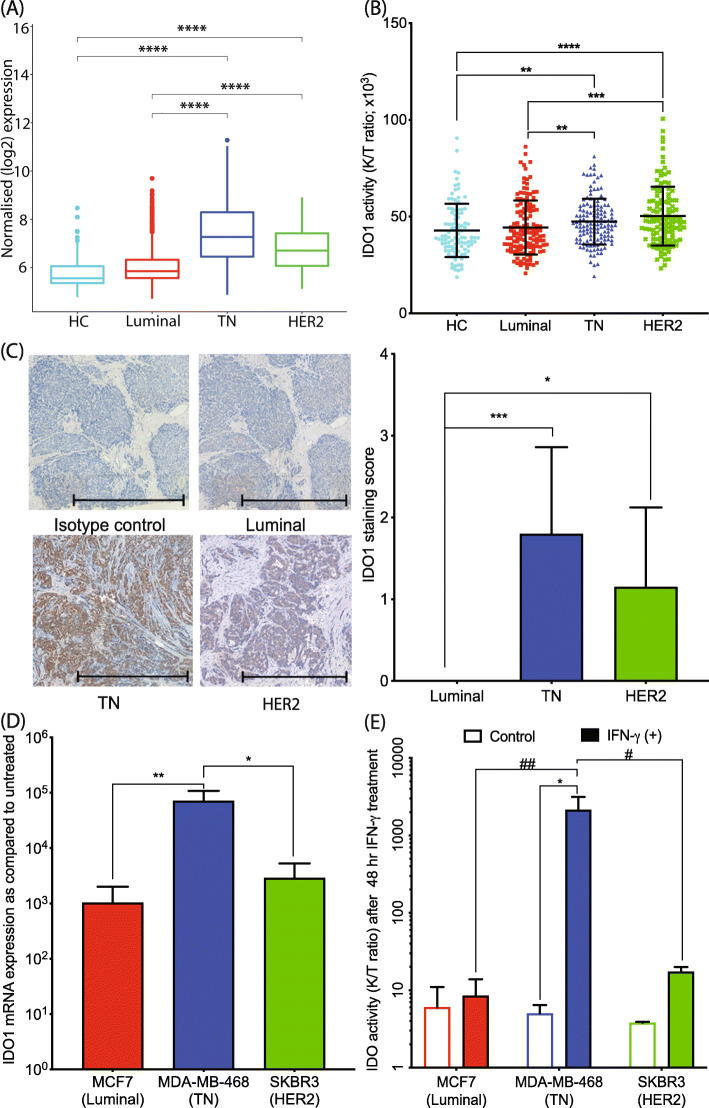
IDO1 enzyme expression and activity in HC and various BrCa subtypes. **a** IDO1 mRNA log2-normalized expression level is significantly increased in TN (*n* = 199) and HER2-enriched (*n* = 220) BrCa subtypes, compared to luminal (*n* = 1140) and healthy control (*n* = 144) tissues from the METABRIC dataset. **b** IDO1 activity (as reflected by the ratio of KYN to TRP concentration, K/T ratio) is upregulated in TN (*n* = 143) and HER2-enriched (*n* = 127) BrCa patient serum relative to HC (*n* = 98) and luminal (*n* = 138) BrCa patient sera. **c** IDO1 protein immunohistochemistry in BrCa tissues shows pronounced IDO1 staining in TN and HER2-enriched BrCa subtypes. Representative images of IDO1 staining in BrCa tumor tissue subtypes with semi-quantification (*n* = 10 each subtype; scale bar, 1000 μM). **d** Induction of IDO1 mRNA is approximately 100-fold higher in TN cells relative to HER2 and luminal BrCa cells after 24 h IFN-γ treatment (*n* = 3, in triplicate). **e** Only TN BrCa cells show a marked translation of active IDO1 protein as judged by K/T ratio after 48 h IFN-γ treatment (*n* = 3, in triplicate). The error bars indicate the standard deviation from the triplicates of cell culture treatment. KP metabolite analysis was performed using uHPLC. *p < 0.05; **p < 0.01; ***p < 0.001; ****p < 0.0001

Given the important role of KP (Fig. 1) in immunosuppression and tumor growth, we screened BrCa models for the first rate-limiting enzyme of the KP, IDO1, which is known to play a significant role in modulating immune response using the publicly available, large population-based METABRIC BrCa dataset. This analysis showed that IDO1 gene expression in BrCa tissue is upregulated in TN and HER2-enriched BrCa subtype compared to luminal BrCa and HC (Fig. 2a). This result was consistent with the serum KYN to TRP concentration ratio (K/T ratio) from cohort 1 showing increased IDO1 activity in both TN (*p* < 0.01) and HER2-enriched (*p* < 0.001) BrCa subtypes (*F*(3, 483) = 1.531, Fig. 2b). Furthermore, assessing the levels of IDO1 protein expression in BrCa tissues from cohort 2 showed that IDO1 was highly expressed in TN and HER2-enriched BrCa subtypes (*F*(2, 27) = 5.52; Fig. 2c) but was absent (or minimally detected) in luminal BrCa, results which were consistent with the serum KP metabolite profiles in cohort 1.

To further extend these findings, we modeled a pro-inflammatory tumor microenvironment by exposing BrCa cell lines to the inflammatory cytokine IFN-γ, one of the most potent IDO1 inducers, at pathophysiologically relevant concentrations for up to 48 h and then assessed the effect on the KP profile. After 24 h exposure, IDO1 gene expression was again highest in the TN BrCa cell line followed by HER2-enriched cell line, SKBR3, then the luminal cell line, MCF7 (Fig. 2d). These results were also consistent with the METABRIC data. Interestingly, post-translational production of active IDO1 enzyme, which occurs by 48 h INF-γ treatment, was indicated by markedly elevated K/T ratio only in the TN cell lines, suggesting that IDO1 activity is only highly inducible in TN BrCa cells in a pro-inflammatory milieu (Fig. 2e and Supplementary Fig. 1A and D).

### KMO activity markedly upregulated in HER2-enriched BrCa subtype

**Fig. 3:**
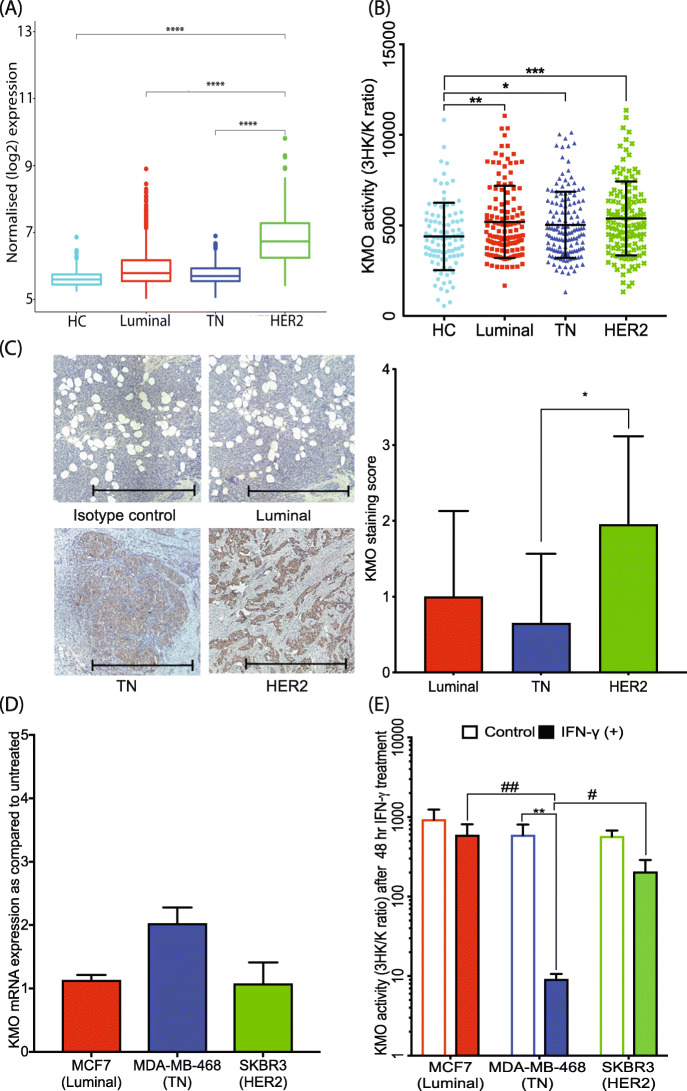
KMO enzyme expression and activity in HC and various BrCa subtypes. **a** All BrCa tissues expressed KMO mRNA more highly than the HC, with HER2-enriched tissues showing most expression in the METABRIC dataset. **b** KMO activity (as reflected by the ratio of 3HK to KYN concentration, 3HK/K ratio) is upregulated in all BrCa patient sera relative to HC, with HER2-enriched BrCa patient serum showing the highest KMO activity. **c** KMO protein immunohistochemistry in BrCa tissues shows that KMO expression is highest in the HER2-enriched BrCa subtype. Representative images of KMO staining in BrCa tumor tissue subtypes with semi-quantification (*n* = 10 each subtype; scale bar, 1000 μM). **d** KMO mRNA expression increased only in TN BrCa cells (2-fold change) after 24 h IFN-γ treatment (*n* = 3, in triplicate). **e** The low 3HK/KYN ratio observed in the TN BrCa cell lines could potentially be due to the elevated production of substrate KYN induced by IFN-γ treatment. KP metabolite analysis was performed using uHPLC in BrCa cell supernatants and cell pellets (*n* = 3, in triplicate) or in human plasma samples, HC (*n* = 98), luminal (*n* = 138), TN (*n* = 143), and HER2-enriched (*n* = 127). The error bars in **d** and **e** indicate the standard deviation from the triplicates of cell culture treatment. *p < 0.05; **p < 0.01; ***p < 0.001; ****p < 0.0001

KYN sits at a branching point of the KP where it can be catabolized into three different intermediates, 3HK, kynurenic acid, or AA by the enzymatic activity of KMO, kynurenine aminotransferase (KATs), or KYNU, respectively. In the METABRIC samples, all BrCa tissue subtypes had higher KMO gene expression levels compared to HC with the highest expression found in the HER2-enriched BrCa subtype (Fig. 3a). The KP metabolite profile of cohort 1 corroborates with the public data, as serum 3HK/KYN ratio, a measure of active KMO enzyme activity, was also highest in the HER2-enriched subtype with all other BrCa subtypes showing elevated KMO relative to the HC group (*F*(3, 491) = 0.5707, Fig. 3b). Immunohistochemical KMO staining was also highest in HER2-enriched BrCa tissues in cohort 2 (*F*(2, 27) = 0.4258; Fig. 3c).

As KMO is inducible during inflammation [29], we also examined KMO expression and activity in different BrCa cell lines after IFN-γ stimulation for 24 or 48 h. KMO mRNA was constitutively expressed with no significant differences between luminal and HER2-enriched cell lines (Fig. 3d). In TN BrCa cells, a twofold increase in KMO mRNA was observed. However, KP metabolite concentrations measured after 48 h of IFN-γ treatment showed that the 3HK/KYN ratio was significantly reduced in TN cells (*p* < 0.01) (Fig. 3e). The 3HK/KYN ratio, while indicative of KMO activity, is also responsive to KYN production or 3HK consumption. As noted above in Fig. 2e, IFN-γ treatment dramatically increased the K/T ratio, i.e., KYN production, in TN cells by inducing IDO1, which may explain these results. The KP enzyme profile of other BrCa cell lines was similar by subtype to the results described above (Supplementary Fig. 1B and E).

### KYNU is highly upregulated in HER2-enriched serum samples with preferential de novo AA synthesis

**Fig. 4:**
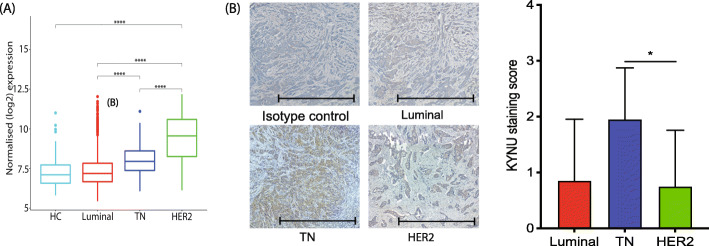
KYNU enzyme expression and activity in HC and various BrCa subtypes. **a** KYNU mRNA expression level is most significantly increased in HER2-enriched BrCa patient tumor tissues from the METABRIC dataset. **b** KYNU protein immunohistochemistry in BrCa tissues shows that KYNU expression is highest in the TN BrCa subtype. Representative images of KYNU staining in BrCa tumor tissue subtypes are shown with semi-quantification (*n* = 10 each subtype; scale bar, 1000 μM)

**Fig. 5:**
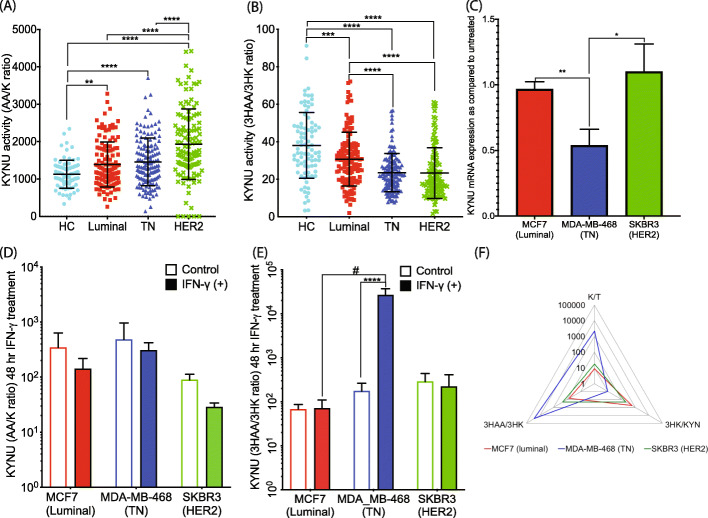
KYNU is dysregulated in HER2-enriched and TN BrCa subtype. **a** KYNU activity along the minor KP sub-branch leading to AA (as reflected by the AA/K ratio) is upregulated in all BrCa patient sera relative to HC, with HER2-enriched BrCa patient serum showing the highest KYNU activity in this sub-branch. **b** KYNU activity along the major KP sub-branch that leads to 3HAA (as reflected by the 3HAA/3HK ratio) is downregulated in all BrCa patient sera relative to HC, with HER2-enriched BrCa patient serum showing the lowest KYNU activity in this sub-branch. **c** KYNU mRNA expression is not induced in TN BrCa cells after 24 h IFN-γ treatment (*n* = 3, in triplicates) relative to untreated cells. **d** KYNU activity along the minor KP sub-branch that leads to AA (as reflected by AA/K ratio) does not significantly change in BrCa cell lines after 48 h IFN-γ treatment, whereas **e** KYNU activity along the major KP sub-branch leading to 3HAA (as reflected by 3HAA/K ratio) is significantly upregulated in TN BrCa cells lines after 48 h IFN-γ treatment. **f** Radar chart of KP enzyme activity after IFN-γ treatment (as reflected by various KP metabolite ratios) shows the distinct pattern of KP dysregulation in TN BrCa cells, relative to luminal and HER2-enriched BrCa cells (increased IDO1 and major sub-branch KYNU activity) which, in turn, leads to the preferential production of the immune-suppressive metabolite 3HAA. KP metabolite analysis was performed using uHPLC in BrCa cell supernatants and cell pellets (*n* = 3, in triplicate) or in human plasma samples, HC (*n* = 98), luminal (*n* = 138), TN (*n* = 143), and HER2-enriched (*n* = 127). The error bars in **c**–**e** indicate the standard deviation from the triplicates of cell culture treatment. *p < 0.05; **p < 0.01; ***p < 0.001; ****p < 0.0001

The KYNU mRNA expression levels in the METABRIC data were highest in HER2-enriched followed by TN subtype relative to luminal and controls (Fig. 4a). Assessing cohort 2 by immunohistochemistry showed that the highest expression of KYNU protein was found in TN, rather than HER2-enriched BrCa tissue (Fig. 4b). This discrepancy between the cohorts could be due to the distribution of sample size in various grades of BrCa. The TN and HER2-enriched samples used in cohort 2 were all grade 3 while the METABRIC cohort consisted of samples from all stages. The KYNU enzyme is located at two different points in the KP and catabolizes two different substrates, KYN and 3HK, leading to the production of AA and 3HAA, respectively (Fig. 1). KP metabolite analysis of cohort 1 plasma showed a much higher AA/KYN ratio in HER2 samples, indicating that KYNU activity is highest in this subtype (*F*(3, 467) = 17.12, Fig. 5a) and also highlighted that the production of AA and not 3HAA is the preferred KP sub-branch. As AA/KYN and 3HAA/3HK ratios are interdependent (AA is produced from KYN by KYNU in one sub-branch but 3HK is converted to 3HAA by KYNU in the other sub-branch), the AA/KYN ratio should show an inverse relationship with the 3HAA/3HK ratio. This was also observed in our clinical plasma samples (Fig. 5b). Possible explanations of the marked difference between AA and 3HAA production among the BrCa subtype include the production of KYN by activated IDO1 in these patients. This could potentially lead to the saturation of KYNU upstream of the pathway by KYN. While increasing AA, KYNU saturation would limit the conversion of 3HK to 3HAA. Additionally, the failure of an oxidation-reduction reaction in the system to convert the AA into 3HAA could also lead to this discrepancy [30]. Another potential explanation leading to a high concentration of AA in the sera is the availability of cofactors such as metal ions and vitamin B6 to KYNU. Deficiency in one of these cofactors has been shown to reduce the activity of the enzyme to produce AA [31].

### Substrate-dependent KP activity leads to de novo 3HAA synthesis in TN BrCa cell line

TN BrCa cell lines treated with IFN-γ did not show a significant increase in KYNU mRNA expression (Fig. 5c and Supplementary Fig. 1C) or in the AA/KYN ratio (Fig. 5d and Supplementary Fig. 1F). Despite these observations, the 3HAA/3HK ratio was highest in TN BrCa cells compared to luminal and HER2-enriched subtype cells (Fig. 5e and Supplementary Fig. 1G) implying an enhanced KYNU activity. The concentration of the 3HAA metabolite increased to 4860 nM compared to 17 nM in non-IFN-γ-treated TN cells (a 286-fold increase). Potential explanations of these results include a higher non-enzymatic conversion of AA to 3HAA or the increased availability of KYN substrate resulting from hyperactivation of IDO1 due to IFN-γ treatment as depicted in the radar chart (Fig. 5f).

### Differential KP profiling discriminates TN from non-TN BrCa cases

Using a supervised machine learning approach, we identified that the most important predictors of TN BrCa were AA/KYN (0.34), age (0.29), 3HAA/AA (0.14), K/T ratio (0.08), TRP (0.08), and KYN (0.07) in our clinical cohort 1 dataset. We then applied classification modeling to KP metabolite concentration and KP enzyme ratios in the BrCa subtypes and control sera. Among the classification models, the C5.0 decision tree was found to have the best accuracy for each BrCa subtype and HC, ranging from 36.7% (control) to 66.4% (TN). These predictors performed poorly in discriminating HC but had better accuracy for TN.

**Fig. 6:**
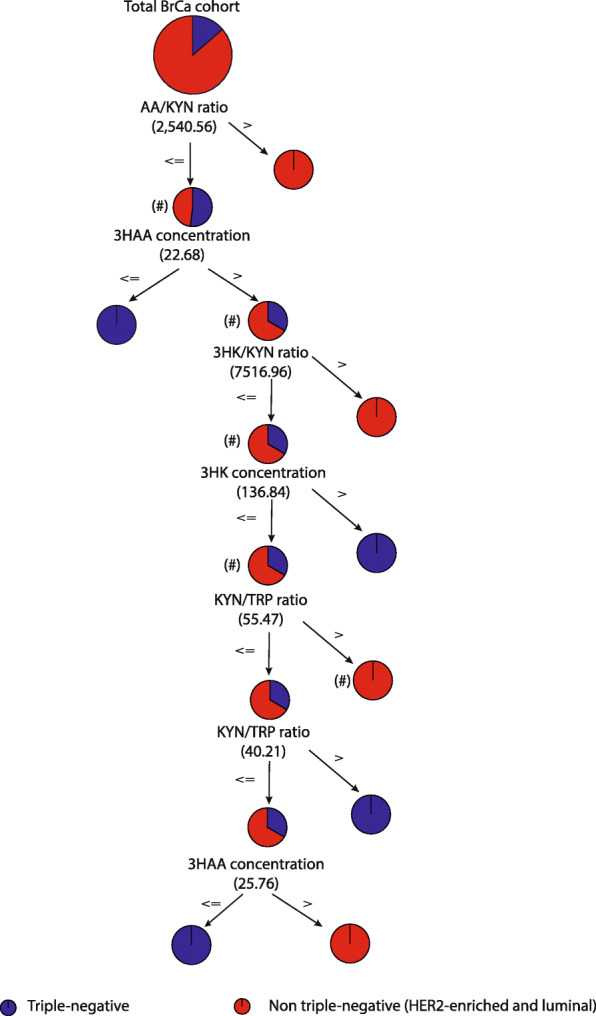
Schematic representation of the C 5.0 decision tree that gave the best model to predict TN BrCa patients from other BrCa patients. The schematic flowchart of the C5 decision tree is being used as a classification model to predict BrCa subtypes. The C5 decision tree is applied to discriminate TN cases from non-TN cases (i.e., luminal and HER2-enriched). The predictors used to sort the cases into various groups were 3HAA, AA/KYN ratio, 3HK, K/T ratio, and 3HK/KYN ratio. This was done in a hierarchical order of importance, calculated by the analytical software (data not shown). The purpose of the model is to completely segregate all the TN cases from the non-TN cases using cutoff values from the computed predictors that are iterated by the C5 decision tree algorithm. For example, a BrCa patient with a AA/KYN ratio of less than or equal to 2540.6 (#), a 3HAA concentration was greater than 22.68 nM (#), a 3HK/KYN ratio was less than or equal to 7516.96 (#), a 3HK was less than or equal to 136.84 nM (#), and a K/T ratio greater than 55.47 (#), then the model predicted them to have non-triple-negative cancer with an accuracy of 95.2%

We then re-trained our training set using BrCa without controls and classified the subtypes into TN and non-TN (luminal and HER2). The new predictors of importance were 3HAA (0.43), AA/KYN (0.23), 3HK (0.15), K/T ratio (0.11), and 3HK/KYN (0.08). Our final model, using a C5.0 decision tree, provided the best subtype prediction for TN with a sensitivity of 95.2% and 63.2% in the training and test sets, respectively (Fig. 6). This model was 44% more accurate than randomly allocating observations to TN or non-TN groups (i.e., lifts for TN BrCa cases and non-TN BrCa cases were 1.4; Fig. 6).

## Discussion

Although the KP has emerged as a key immunotherapeutic target for cancer, the role of this pathway in BrCa has been relatively understudied. We have sought to fill this gap by comprehensively examining the KP in each major BrCa subtype, which has resulted in some novel findings. Our first key finding is that the KP is more highly dysregulated in the aggressive HER2-enriched and TN BrCa subtypes. Our second key finding is that the enzymes KMO and KYNU are highly upregulated in the aggressive HER2-enriched and TN BrCa subtypes. These results possibly provide a link between highly proliferative, metastatic lesions and KP dysregulation, as excessive KMO or KYNU activity increases the production of immunosuppressive metabolites. Our clinical and in vitro data has also confirmed the important role played by IDO1, particularly in the TN subtype, in producing excess KYN that fuels the KP downstream. We have also observed, for the first time, that TN BrCa cells are capable of producing the immunosuppressive KP metabolite 3HAA. Significantly, our study shows that serum KP profiles can successfully discriminate TN BrCa patients from other BrCa patients.

Our data has demonstrated that clinical samples from TN BrCa patients had much higher IDO1 expression. We hypothesized that a number of upstream genes associated with the TN BrCa subtype could be upregulating IDO1 expression. In particular, gene profiling of TN breast tumors revealed that STAT3 and NF-κB were highly activated. These two genes have been shown to crosstalk, potentiating IDO1 upregulation in myeloid-derived suppressor cells [32, 33]. Yu et al. further demonstrated in a mouse model that the disruption of the STAT3–NF-κB–IDO1 axis using a small-molecule inhibitor of STAT3 led to a significant downregulation of IDO1 expression in tumor-suppressing myeloid cells and a reduction in the size of BrCa tumors [34]. Another potentially significant gene cascade is the cytokine signaling 3 suppressor (SOCS3) [35], one of the major negative feedback regulators of inflammatory cytokine signaling pathways such as the JAK/STAT signaling pathway [36] and a known suppressor of IDO1 [37, 38]. TN BrCa has low expression of SOCS3. Fallarino et al. and Pallotta et al. have shown that the induction of *SOCS3* in IDO1-positive dendritic cells inhibits IDO1 transcription [37] and targets IDO1 protein for proteasome degradation [38]. Hence, low SOCS3 expression is a probable factor in the enhanced IDO1 expression seen in TN breast tumors. High expression of the aryl hydrocarbon receptor (AhR) in TN BrCa may provide another compelling explanation of high IDO1 expression. AhR is a master environment-responsive immune regulator, which has been found to be highly expressed in TN BrCa cells [39, 40]. AhR not only directly increases IDO1 expression [41] but is also responsible for enzymatic IDO1 phosphorylation during post-translational modification [42]. It is possible to speculate that the above genes may work in tandem to shift the balance towards the induction of IDO1 expression in TN BrCa.

Our in vitro data (Fig. 2d) have shown that the TN BrCa cell line, MDA-MB-468, has the highest IDO1 expression compared to cell lines of other BrCa subtypes. We have also found that induction of IDO1 by IFN-γ results in markedly increased KYN production in TN BrCa cell lines, but not in other BrCa cell lines relative to the untreated control (Fig. 2e and Supplementary Fig. 1D). Considering that KYN is an AhR ligand [43], this suggests a possible autocrine feedback loop between AhR and IDO1 that impairs immune system function, particularly in TN BrCa.

Crucially, our finding that high IDO expression is specific to TN and not other BrCa subtypes may partially explain the limited efficacy of IDO1 inhibitors in clinical trials. The 2018 phase 3 clinical trial (NCT02752074) that examined the effectiveness of combining an IDO1 inhibitor, epacadostat, with the checkpoint inhibitor pembrolizumab found that melanoma patient progression-free survival was not improved. Other clinical trials have successfully used patient pre-treatment screening to ensure an “on-target” treatment cohort. For example, anti-PD-L1 immunohistochemistry screening identified lung cancer patients most likely to benefit from pembrozlizumab treatment [44]. Considering the success of this screening strategy, the inclusion of KP profile screening of cancer patients may improve treatment efficacy in future trials of IDO1 inhibitors. More specifically, our study suggests that TN BrCa patients are most likely to benefit in future clinical trials involving IDO1 inhibitors.

Despite the focus on IDO1, there is growing evidence demonstrating that IDO1 is not the only KP enzyme that produces immune modulatory metabolites. In particular, the downstream KP metabolites 3HK and 3HAA have been shown to modulate T cell populations. Both Fallarino et al. and Zaher et al. have reported that 3HK inhibits CD4+ T cell proliferation [12, 13], and Zaher et al. have further shown that 3HK promoted the proliferation of CD25+ T cells [13]. Another potent immunosuppressive KP metabolite, 3HAA, has also been shown to inhibit CD8+ T cell proliferation [45]. In addition to the immunotoxic effect on T cell proliferation, Platten et al. have shown that 3HK and 3HAA can inhibit the production of pro-inflammatory cytokines by CD4+ T cells [46]. Our study has shown that the 3HK-producing enzyme, KMO, is highly elevated in BrCa, especially in HER2-enriched BrCa tissue, concordant with the high serum level of 3HK detected in people with BrCa (Fig. 3a–c). In agreement with our study, the global quantitative proteomic mapping study examining BrCa subtypes identified KMO as a significantly upregulated protein in HER2-enriched BrCa subtype. The molecular mechanisms underlying enhanced KMO expression in HER2-enriched, however, need further investigation.

Also, elevated in HER2-enriched BrCa tissue was the enzyme KYNU, which produces both 3HAA and AA. Our ex vivo metabolite profiling data further revealed that KYNU preferentially produces AA over 3HAA, suggesting that downstream KP metabolites may have a role in BrCa aggressiveness. To address whether the downstream activity of KMO and KYNU results from IDO1 induction, we examined de novo KP metabolite biosynthesis following IDO1 induction by IFN-γ in BrCa cell lines. These data showed that luminal and HER2-enriched BrCa cells share a similar profile favoring 3HK production, whereas TN cells have a preference for 3HAA production, as illustrated by the radar chart (Fig. 5f). Considering the potent immune-suppressive effects of 3HAA, this could be a significant factor in the relative aggressiveness and metastatic potential of TN BrCa. High 3HAA production may also account for the low levels of 3HK in TN BrCa cells (Fig. 3e) as 3HK can be rapidly metabolized to 3HAA (Fig. 5f).

While our in vitro data shows a higher 3HAA concentration in TN BrCa cells, the concentration of 3HAA was lower in TN BrCa patient sera. Potential reasons that may explain this include the fact that 3HAA is a highly reactive compound that is auto-oxidized more rapidly, as compared to AA in aqueous conditions (serum) [47]. Additionally, the conversion of AA to 3HAA is dependent on milieu pH and iron availability [48], and it is interesting to note that the aggressiveness of the TN subtype has been shown to be related to the balance of iron-regulating genes that lead to tumor iron accumulation [49]. Considering that both TN and HER2-enriched BrCa feature a more dysregulated and immunosuppressive KP compared to the less aggressive luminal subtype, it would be interesting to assess the inhibition of KMO and KYNU, which lead to AA and 3HAA, respectively, in animal models of TN breast cancer.

The application of classification modeling for biomarker discovery has become increasingly popular. In BrCa, serum TRP and KYN levels have been suggested as biomarkers for monitoring treatment efficacy [18, 50]. Hence, we explored if our extensive BrCa KP profiling data may have additional biomarker potential for clinical translation. Using an unbiased machine learning approach, we initially attempted to discriminate BrCa subtypes from HC. We observed that KP metabolites were not able to discriminate HC from BrCa cases because the KP can be dysregulated by many inflammatory conditions. Hence, we developed models with a prognostic focus that excluded healthy controls as potential confounders. Our final model (Fig. 6) demonstrated a strong discriminating ability to identify TN BrCa with up to 95.2% accuracy, which approximates the sensitivity achieved by current clinically used biomarkers to identify luminal and HER2-enriched BrCa patients (93.7–97% and 80–96.2%, respectively) [51, 52]. Interestingly, age, a well-known risk factor for BrCa, was no longer an important factor when predicting BrCa subtype, highlighting the applicability of using KP predictors in assessing BrCa prognosis and also suggesting a potentially dependent relationship between the KP and BrCa pathology, independent of age. This approach demonstrates the value of KP as a biomarker for targeted therapies in breast cancer and as a surrogate marker for intrinsic subtype prediction.

Our study has limitations. As a cross-sectional study, it is not possible to assess KP metabolites in terms of TN risk or survival that would be possible with a longitudinal cohort. Such data could strengthen the case that downstream KP enzymes, KMO and KYNU in particular, play a significant role in mediating BrCa aggressiveness, thereby suggesting new potential therapeutic approaches in BrCa. As the HC group in our clinical cohort was significantly younger compared to BrCa groups, age might have been a confounding factor. However, as discussed above, our unbiased machine learning method indicated that age was not a significant factor in discriminating the TN subtype. Finally, the lack of matching immune profile data limits our ability to assess the interplay between the immune system and the KP in our clinical cohorts.

## Conclusion

We have demonstrated, for the first time, that the downstream KP enzymes KMO and KYNU are hyperactive in more aggressive BrCa subtypes, leading to enhanced production of the immunosuppressive metabolites AA and 3HAA. Thus, we have demonstrated that the well-described role played by the KP in mediating cancer immune tolerance extends beyond IDO1, potentially identifying KMO and KYNU inhibitors as new therapeutic BrCa targets. Our data further suggests that KP metabolite profiling has potential as a biomarker in BrCa subtyping, as our study successfully discriminated TN BrCa from other BrCa subtypes.

## Supplementary information

**Additional file 1: Figure S1.** IDO1, KMO and KYNU expression in luminal and TN BrCa cell lines. IDO1, KMO and KYNU mRNA expression were quantified using qPCR after after 24 hrs IFN-γ treatment (*n* = 3, in triplicate). a IDO1 mRNA expression is highly elevated in TN BrCa cell lines by an approximately 10-fold higher as compared to luminal BrCa cell lines. b KMO mRNA expression is induced in luminal BrCa cell line (approximately 2-fold change) and TN BrCa cell lines (approximately 10-fold change) c KYNU mRNA expression increased only in TN BrCa cell lines except MDA-MB-157 (approxximately to 1.5 to 2-fold change). d Only TN BrCa cell lines showed singificant IDO1 activity as reflected by K/T ratio after 48 hrs IFN-γ treatment. e There is a marked decrease in KMO activity in TN BrCa cell lines after 48 hrs IFN-γ treatment as shown by decreased 3HK/K ratio after 48 hrs IFN-γ treatment. However, there is no difference in KMO activity between the IFN-γ treatment and control of luminal BrCa cell line. f There is no difference in the KYNU activity along the minor KP sub-branch that leads to AA (as judged by AA/K ratio) in BrCa cell lines after 48 hrs IFN-γ treatment whereas, g KYNU activity along the major KP sub-branch leading to 3HAA (as shown by 3HAA/K ratio) is upregulated in TN BrCa cells lines after 48 hrs IFN-γ treatment. KP metabolite analysis was performed using uHPLC. * and #, *p*<0.05; ** and ##, *p*<0.01; *** and ###,, *p*<0.001; **** and ####, *P*<0.0001.

**Additional file 2: Table S1.** Realtime PCR primer details.

## Data Availability

The METABRIC microarray dataset used in this study is available at the European Genome-Phenome Archive at http://www.ebi.ac.uk/ega, under the accession number EGAS00000000083 [22]. All remaining data and materials are available from the authors upon reasonable request.

## Abbreviations

BrCa: Breast cancer
KP: Kynurenine pathway
TN: Triple-negative
IDO1: Indoleamine-2,3-dioxygenase 1
TRP: Tryptophan
3HK: 3-Hydroxykynurenine
AA: Anthranilic acid
3HAA: 3-Hydroxyanthranilic acid
KYN: Kynurenine
KMO: Kynurenine monooxygenase
HC: Healthy control
METABRIC: Molecular Taxonomy of Breast Cancer International Consortium
KATs: Kynurenine aminotransferase
KYNU: Kynureninase
SOCS3: Cytokine signaling 3 suppressor
AhR: Aryl hydrocarbon receptor
