# Supplementary figures and images for: Differential kynurenine pathway metabolism in highly metastatic aggressive breast cancer subtypes: beyond IDO1-induced immunosuppression
Source: Breast Cancer Res. 2020 Oct 27;22:113. doi: 10.1186/s13058-020-01351-1 (PMC7590459; doi:10.1186/s13058-020-01351-1)

Supplementary fig. 1

(A)

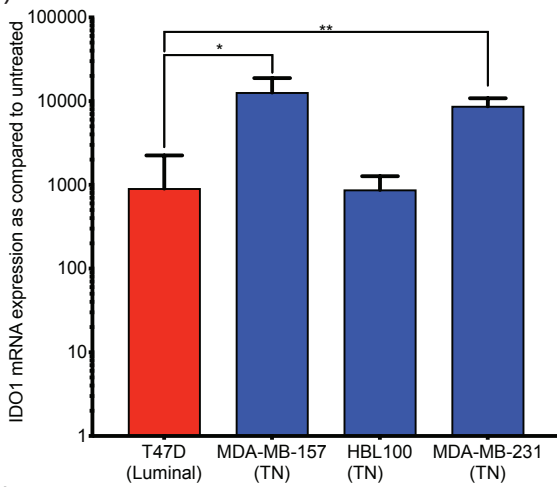

(B)

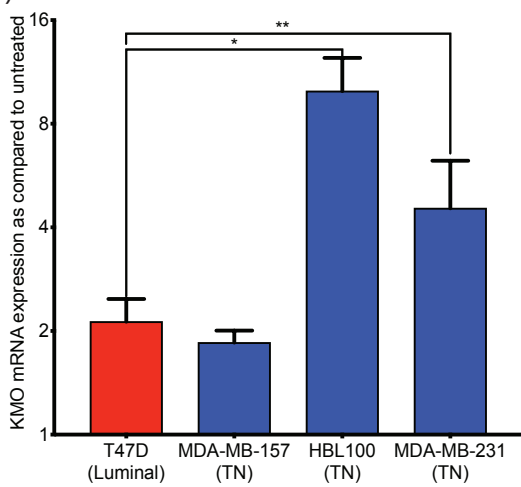

(C)

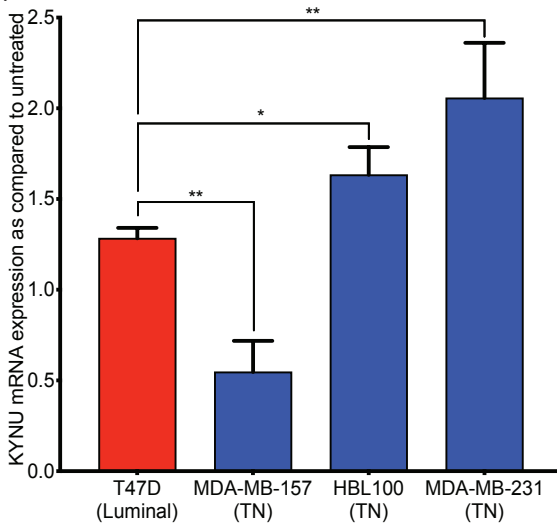

(D)

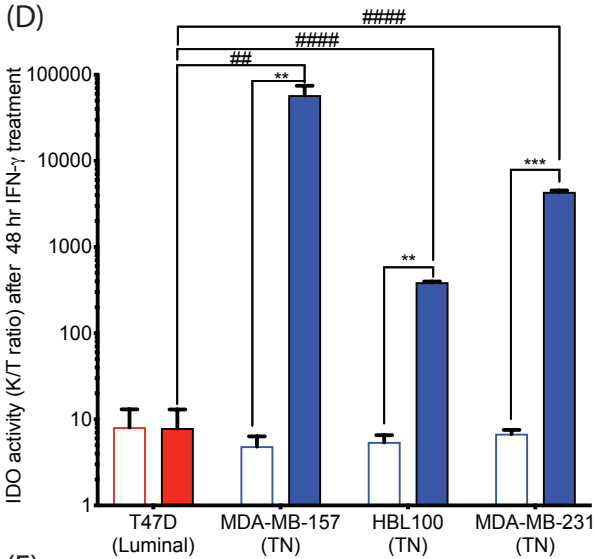

(E)

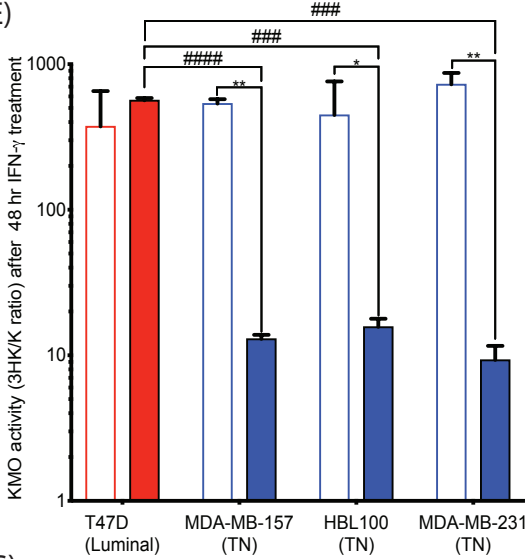

(F)

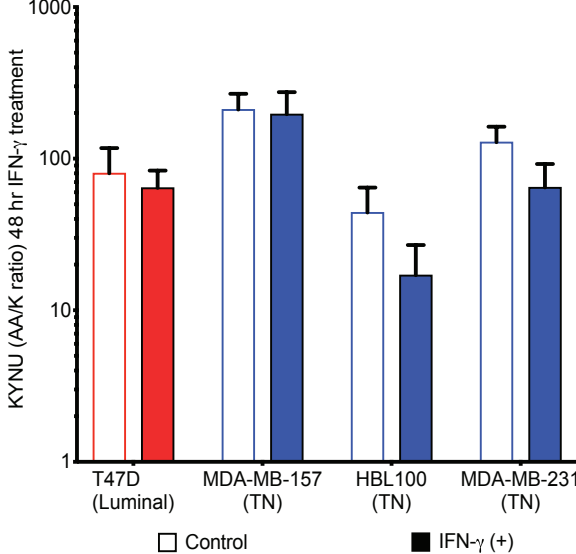

(G)

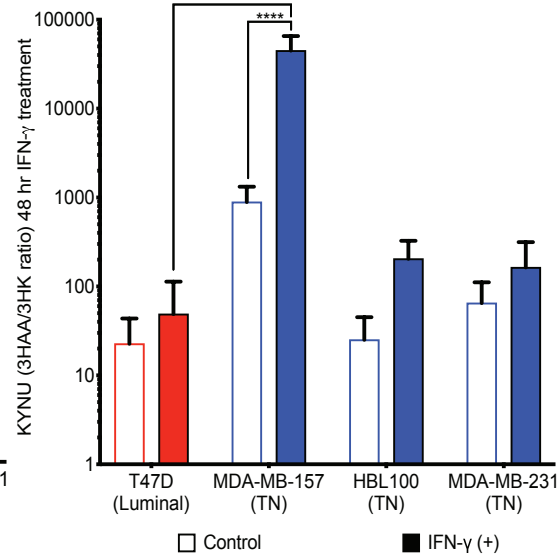

Supplement: Supplementary file 1 — Additional file 1: Figure S1. IDO1, KMO and KYNU expression in luminal and TN BrCa cell lines. IDO1, KMO and KYNU mRNA expression were quantified using qPCR after after 24 hrs IFN-γ treatment (n = 3, in triplicate). a IDO1 mRNA expression is highly elevated in TN BrCa cell lines by an approximately 10-fold higher as compared to luminal BrCa cell lines. b KMO mRNA expression is induced in luminal BrCa cell line (approximately 2-fold change) and TN BrCa cell lines (approximately 10-fold change) c KYNU mRNA expression increased only in TN BrCa cell lines except MDA-MB-157 (approxximately to 1.5 to 2-fold change). d Only TN BrCa cell lines showed singificant IDO1 activity as reflected by K/T ratio after 48 hrs IFN-γ treatment. e There is a marked decrease in KMO activity in TN BrCa cell lines after 48 hrs IFN-γ treatment as shown by decreased 3HK/K ratio after 48 hrs IFN-γ treatment. However, there is no difference in KMO activity between the IFN-γ treatment and control of luminal BrCa cell line. f There is no difference in the KYNU activity along the minor KP sub-branch that leads to AA (as judged by AA/K ratio) in BrCa cell lines after 48 hrs IFN-γ treatment whereas, g KYNU activity along the major KP sub-branch leading to 3HAA (as shown by 3HAA/K ratio) is upregulated in TN BrCa cells lines after 48 hrs IFN-γ treatment. KP metabolite analysis was performed using uHPLC. * and #, p<0.05; ** and ##, p<0.01; *** and ###,, p<0.001; **** and ####, P<0.0001. [file 13058_2020_1351_MOESM1_ESM.pdf]
