# Supplementary material for: Differential kynurenine pathway metabolism in highly metastatic aggressive breast cancer subtypes: beyond IDO1-induced immunosuppression
Source: Breast Cancer Res. 2020 Oct 27;22:113. doi: 10.1186/s13058-020-01351-1 (PMC7590459; doi:10.1186/s13058-020-01351-1)
Supplement: Supplementary file 2 — Additional file 2: Table S1. Realtime PCR primer details. [file 13058_2020_1351_MOESM2_ESM.pdf]

Table 1. Realtime PCR primer details

Primer name	Primer sequence	Annealing temperature/efficiency
IDO1 forward	GCCAGCTTCGAGAAAGAGTTG	55°C / 94%
IDO1 reverse	TGACTTGTGGTCTGTGAGATGA	
KMO forward	TAGCCCTTTCTCATAGAGGACG	55°C / 99%
KMO reverse	CTCTCATGGGAATACCTTGGGA	
KYNU forward	GGCTCTCCACCTAGATGAGGA	60°C / 87%
KYNU reverse	GCTGCTATTTTGGCCCACTTAT	
3-hydroxyanthranilate 3,4-dioxygenase (3HAO) forward	TATGCCTGGGAGCGAACACA	60°C / 99%
3HAO reverse	GCAGGAGGGTGGGTGACAAC	
Quinolinic acid phosphoribosyltransferase forward	GGTCCTGAGCAGCCAACACA	60°C / 89%
QPRT reverse	GAAAGGCTGCCCTGCCAGTA	
TATA-box-binding protein (TBP) forward	GGGAGCTGTGATGTGAAGT	60°C / 95%
TBP reverse	GGAGGCAAGGGTACATGAGA	
Phosphoglycerate kinase 1 (PGK1) forward	TCACTCGGGCTAAGCAGATT	60°C / 90%
PGK1 reverse	CAGTGCTCACATGGCTGACT	
Glucose-6-phosphate dehydrogenase (G6PDH) forward	CCAAGCCCATCCCCTATATT	60°C / 90%
G6PDH reverse	GAATGTGCAGCTGAGGTCAA	
